# Public Health Fallout From Transboundary Sewage Exposure in the Tijuana River Valley

**DOI:** 10.1002/puh2.70203

**Published:** 2026-02-27

**Authors:** Nam T. Nguyen, Jae‑Hee Bae, Dhroov Pathare, Rain Wong, Kevan Shah, Vi T. Nguyen

**Affiliations:** ^1^ University of California San Diego California USA; ^2^ Yale University New Haven Connecticut USA; ^3^ University of Texas at Austin Austin Texas USA; ^4^ University of California Los Angeles California USA; ^5^ Cooper Medical School of Rowan University Camden New Jersey USA; ^6^ Southern California Permanente Medical Group San Diego California USA

**Keywords:** Mexico, sewage, Tijuana River Valley, US

Since 2018, an estimated 100 billion gallons of sewage and industrial contaminants have flowed from Tijuana, Mexico, into the binational Tijuana River Valley, raising substantial public health concerns in adjacent communities on both the Mexican and US sides of the border. This ongoing environmental conflict has been driven by chronic failures in wastewater management and is compounded by infrastructure inadequacies. Residents and environmental advocates have long raised concerns about this issue, with recent multi‐institutional investigations (e.g., the 2024 San Diego State University [SDSU] aerosol‐pathogen study) now providing data on exposure pathways and community‐reported health burdens [1, 2]. This commentary provides a narrative synthesis of publicly available public health assessments and peer‐reviewed environmental studies describing exposure pathways and health concerns related to transboundary sewage contamination in the Tijuana River Valley.

In a community health assessment, the Centers for Disease Control and Prevention's (CDC) 2025 Community Assessment for Public Health Emergency Response (CASPER) noted that about 70% of the surveyed households in the San Diego region reported health symptoms that were associated with sewage exposure (e.g., gastrointestinal illness, skin infections, and respiratory conditions) [1]. Similarly, these acute illnesses align with clinical patterns described by local healthcare providers, although formal surveillance has not yet detected clear spikes in sewage‐related diagnoses. Moreover, peer‐reviewed environmental sampling studies have detected that sewage‐associated microbes can be transported in coastal aerosols, and highlighted viruses such as norovirus and hepatitis A as key pathogens of concern in contaminated coastal waters, suggesting that exposure pathways have extended beyond water contact to also airborne transmission [2, 3]. To date, publicly available clinical surveillance has not produced sewage‐attributable incidence rates, time trends, or effect estimates, highlighting an important gap for future epidemiologic monitoring.

Airborne exposure through sewage derived pathogens has become increasingly recognized as a public health concern in the region. Researchers from SDSU and collaborating institutions have identified high levels of enteric bacteria and respiratory pathogens, including antibiotic‐resistant strains, in aerosol samples and wastewater‐associated chemicals collected near sewage‐impacted coastal waters along the Tijuana River and Imperial Beach [2, 4].

Studies using gene sequencing and mass spectrometry have also found evidence of aerosolized coastal water pollution reaching far beyond communities in direct coastal lands. Elevated concentrations of hydrogen sulfide and other volatile organic compounds may pose additional respiratory hazards, specifically for children and individuals with pre‐existing respiratory conditions [5, 6]. Chemical tracing has confirmed that high wastewater flows and low winds increase hydrogen sulfide contamination of air quality and correlates these peaks with community malodor reports [2, 7].

Residents have also reported instances of persistent cough, wheezing, and other respiratory symptoms in association with exposure episodes [1]. This evidence has underscored a need to increase regional air‐quality monitoring in coastal areas such as Imperial Beach, and to expand targeted public advisories by organizations such as the San Diego County Air Pollution Control District or the Environmental‐Justice Workgroup [8].

Beyond the respiratory conditions, there has been a growing concern regarding potential long‐term health consequences associated with chronic sewage exposure. For instance, environmental assessments by regional public health departments have detected elevated concentrations of heavy metals such as arsenic, lead, and cadmium in riverbanks, recreational parks, and residential regions [2, 9]. These metals are widely recognized toxicants associated with cancer, renal dysfunction, and neurological impairments. Soil sediment analysis has also revealed over 170 chemicals and inorganic elements that include carcinogens such as chlordane, dichlorodiphenyltrichloroethane (DDT), phthalates, and polycyclic aromatic hydrocarbons (PAHs) [2]. However, available sources do not consistently report contaminant concentrations relative to health‐based benchmarks, limiting quantitative risk characterization and underscoring the need for standardized reporting against established exposure standards.

The psychological burden has long affected the residents of the region. In the 2025 CASPER survey, 59% of the residents experienced heightened stress levels, 56% reported sleep disturbances, and 38% indicated increased anxiety due to the ongoing sewage crisis [1]. The consequences of the Tijuana River Sewage crisis have exacerbated mental‐health issues with patterns mirrored in other onset disasters, such as the Flint Water Crisis in Michigan, where the prolonged uncertainty and neglect aggravated local resident psychological trauma [10, 11]. Both socioeconomic and political vulnerabilities, such as floods contaminating and washing away homes, have also compounded mental health distress.

Local healthcare infrastructure has been increasingly strained by the ongoing health impacts associated with sewage contamination. Although many households report symptoms they attribute to sewage exposure, relatively few seek formal medical care, and existing clinic and urgent‐care surveillance has not yet detected clear increases in sewage‐related diagnoses. Consequently, the economic and health‐system burdens have been under‐reported and under‐addressed, limiting knowledge of the full‐scale public health crisis [1, 2].

To address the Tijuana River sewage crisis, coordinated policy actions are warranted to reduce exposures, strengthen monitoring, and mitigate community impacts. First, policymakers should invest in significant wastewater infrastructure to expand treatment capacity, improve sewage containment during peak‐flow events, and strengthen cross‐border collaboration with Mexico on infrastructure oversight. Second, strengthened health surveillance systems are needed to detect both short and long‐term health risks from the Tijuana River; this would include biomonitoring of environmental toxicology, zoonotic diseases, and microbial pathogens. Third, it is essential to provide public health agencies and community partners, such as San Diego County Air Pollution Control District and the Environmental Justice Workgroup (EJW), with greater resources and authority to better inform communities about the ongoing exposure risks in San Diego. Finally, psychological impacts must be recognized as one of the primary consequences to the Tijuana River sewage crisis and be included in the response framework.

## Author Contributions

Nam T. Nguyen: Conceptualization, Investigation, Writing – original draft, Writing – review & editing. Jae‐Hee Bae: Investigation, Writing – review & editing. Dhroov Pathare: Investigation, Writing – review & editing. Rain Wong: Investigation, Writing – review & editing. Kevan Shah: Investigation, Writing – review & editing. Vi T. Nguyen: Supervision, Conceptualization, Writing – review & editing. All authors read and approved the final manuscript.

## Funding

The authors have nothing to report.

## Conflicts of Interest

The authors declare no conflicts of interest.

## Data Availability

Data sharing is not applicable to this article as no datasets were generated or analyzed during the current study.
